# Roswell Park

**Published:** 1914-03

**Authors:** 


**A Monthly Review of Medicine and Surgery**

EDITOR AND PUBLISHER DR. A. L. BENEDICT, 228 Summer St., corner of Elmwood Ave., Buffalo. (Address for all communications. Please make personal and telephone calls before 1 P. M.)

Third, in age, in the western continent. Independent but supports professional organization. News limited mainly to western N. Y. and Penn.

Subscription $2.00 a year; $3.00 for two years in advance. Changes and errors in address or failure or duplication of delivery, should be reported immediately.

YEARLY VOLUME 69 MARCH, 1914 No. 8

### Roswell Park

In addition to the more extensive description of Dr. Park's life and work in other departments, we feel that his impress upon the profession generally, was such that he must be considered as a factor, as well as an individual. After an acquaintance of nearly thirty years, beginning in a boy's hero-worship, continued as a student, and later, in the frequent contacts of professional, club and other associations, it is difficult to eliminate personal sentiment, but justice demands that this impersonal force of Dr. Park's activities should be recognized. The morning following Dr. Park's death, we received with what emotions may be imagined, a letter from him—his very last one, as we learned later—delegating certain duties on a committee and containing these words which, in the light of the sad event, are tinged with the irony of fate: "Within two or three days I hope to be able to get away for a much needed rest of somewhat indefinite length." In 1897, Dr. Park was thought to be fatally ill, due to the remote results of a diphtheritic and of a subsequent septic infection, contracted at operations. This ordeal was a handicap from which he never fully recovered in the physical sense. Yet, in looking back over the nearly seventeen years, we must recognize that he did his best work in spite of it and, within a week or two of his death, he spoke so vigorously, almost sternly, of the general proposition of retirement, that we must recognize that the words quoted were prophetic only by a sad coincidence and that his rest was intended merely to prepare him for future hard work.

In the face of death all men are equal, and, in its appreciation of the men who have gone from it after lives of diligence and probity, the medical profession is no respector of persons. Yet the dead would, as freely as the living, concede that of all men who have completed their life work in our profession in Buffalo, Roswell Park has achieved the highest degree, not merely of eminence, but of greatness.

